# Researches as to the Value of Mallein as a Diagnostic Agent in Glanders

**Published:** 1898-06

**Authors:** Alexander Glass

**Affiliations:** Philadelphia


### FRENCH.

UNDER THE DIRECTION OF DR. ALEXANDER GLASS,
PHILADELPHIA.

RESEARCHES AS TO THE VALUE OF MALLEIN AS A DIAGNOSTIC AGENT IN GLANDERS.—In a stable containing forty-six horses, four developed and subsequently died from acute glanders. Not only was the disease diagnosticated during the life of the animals, but after death, postmortem being made. By order of the authorities the remaining forty-two were condemned and used to demonstrate the value of mallein as a diagnostic agent in future suspected cases of glanders.

On March 18th, at noon, the injections were commenced, the temperatures having been taken before any injections were made; observations were also made two hours, five hours, and fourteen hours after the injections. On the first day eight of the horses were injected ; the second day, twenty ; and the third day, fourteen. For this observation Drs. Tiede and Olt used mallein obtained from the Prussian veterinary department, and in their report divided the cases into three groups, according to the reaction shown in their temperature after injection.

In nine horses the temperature rose 1.5° ; in the second group, six horses, the temperature rose from 1° to 1.4° ; and in the last group, which consisted of the remaining animals, there was little or no reaction—if there was any, it was so slight as to be hardly worth notice.

The animals were killed a few days afterward, and the results of the postmortems compared with the animals that reacted. Of the forty-two subjects only three were found to present distinct lesions of glanders, and these were in the group of animals that had not presented any reaction at all ; while in those which reacted no actual lesions of the disease were found.

The observers had, therefore, to agree with the opinion of Schultz and others, when they made their reports to the authorities, that the mallein prepared in Prussia could not be regarded as a reliable agent in the diagnosis of glanders in the horse, and while they came to this conclusion in reference to the mallein prepared in Prussia, they do not place it on the same status as that prepared at the Pasteur Institute, which has given great satisfaction, up to the present time, in all parts of the world.—*Archiv fur Wissenschaftliche.*

